# Supplementary material for: Predicting visual function by interpreting a neuronal wiring diagram
Source: Nature. 2024 Oct 2;634(8032):113–23. doi: 10.1038/s41586-024-07953-5 (PMC11446822; doi:10.1038/s41586-024-07953-5)
Supplement: Supplementary file 6 — CRF and ERF predictions for individual TmY4 and TmY9 cells. Analogous to Supplementary Data 3, but for TmY target types. Shown are the top four monosynaptic pathways, the strongest pathway passing through each of the top ten intermediary types (ranking from Extended Data Fig. 7), and the trisynaptic pathway Tm1–TmY–Dm3–TmY (see the section entitled Prediction of spatial normalization). [file 41586_2024_7953_MOESM6_ESM.zip › DataS4/TmY9q/720575940627733013.pdf]

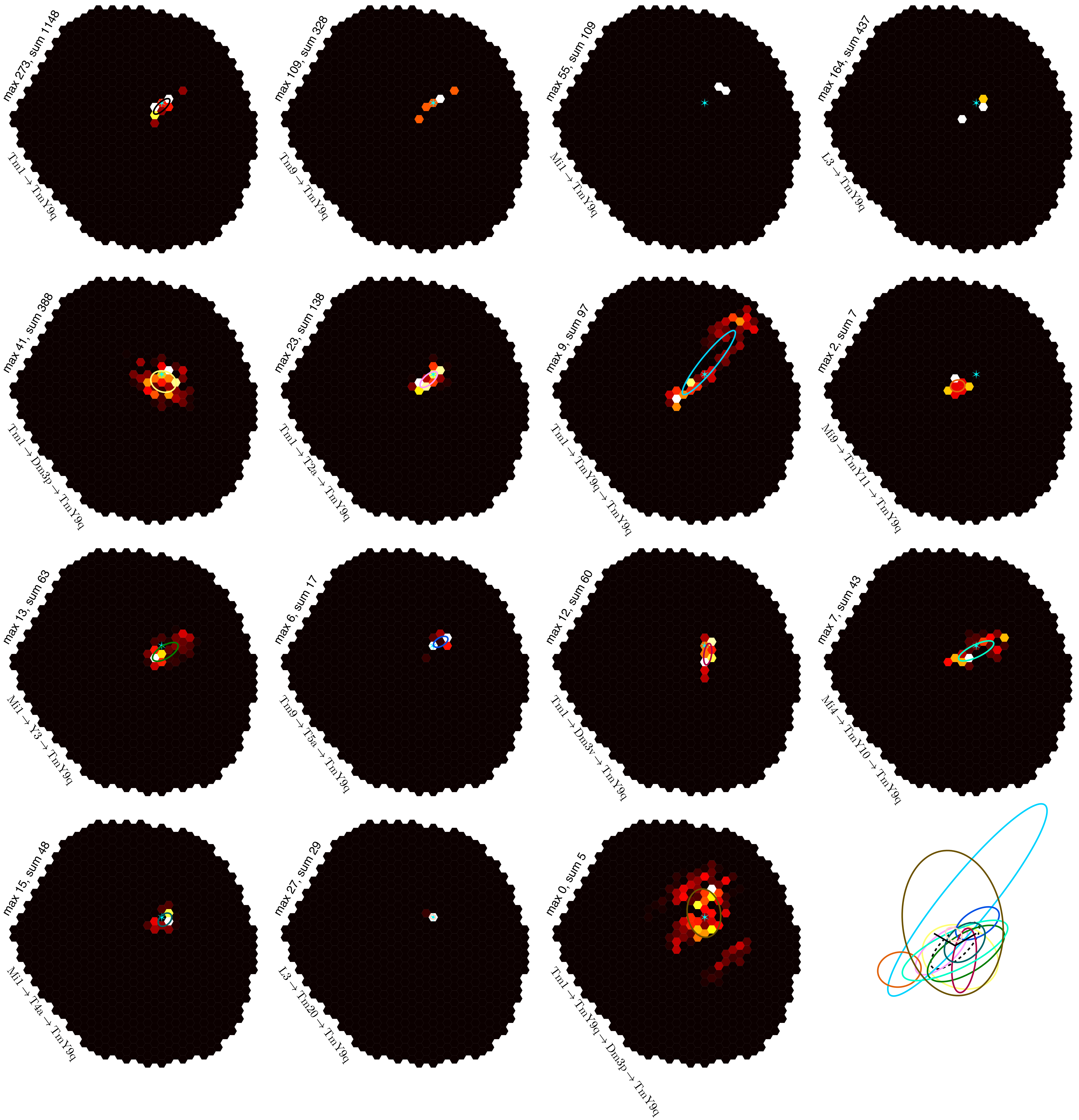
max 273, sum 1148
Tm1 → TmY9q
max 109, sum 328
Tm9 → TmY9q
max 55, sum 109
Mi1 → TmY9q
max 164, sum 437
L3 → TmY9q
max 41, sum 388
Tm1 → Dm3p → TmY9q
max 23, sum 138
Tm1 → T2a → TmY9q
max 9, sum 97
Tm1 → TmY9q → TmY9q
max 2, sum 7
Mi9 → TmY11 → TmY9q
max 13, sum 63
Mi1 → Y3 → TmY9q
max 6, sum 17
Tm9 → T5a → TmY9q
max 12, sum 60
Tm1 → Dm3v → TmY9q
max 7, sum 43
Mi4 → TmY10 → TmY9q
max 15, sum 48
Mi1 → T4a → TmY9q
max 27, sum 29
L3 → Tm20 → TmY9q
max 0, sum 5
Tm1 → TmY9q → Dm3p → TmY9q
